# A Fungal Pathogen of Amphibians, *Batrachochytrium dendrobatidis*, Attenuates in Pathogenicity with *In Vitro* Passages

**DOI:** 10.1371/journal.pone.0077630

**Published:** 2013-10-10

**Authors:** Penny F. Langhammer, Karen R. Lips, Patricia A. Burrowes, Tate Tunstall, Crystal M. Palmer, James P. Collins

**Affiliations:** 1 School of Life Sciences, Arizona State University, Tempe, Arizona, United States of America; 2 Department of Biology, University of Maryland, College Park, Maryland, United States of America; 3 Department of Biology, University of Puerto Rico, San Juan, Puerto Rico, United States of America; Imperial College Faculty of Medicine, United Kingdom

## Abstract

Laboratory investigations into the amphibian chytrid fungus, *Batrachochytrium dendrobatidis* (Bd), have accelerated recently, given the pathogen’s role in causing the global decline and extinction of amphibians. Studies in which host animals were exposed to Bd have largely assumed that lab-maintained pathogen cultures retained the infective and pathogenic properties of wild isolates. Attenuated pathogenicity is common in artificially maintained cultures of other pathogenic fungi, but to date, it is unknown whether, and to what degree, Bd might change in culture. We compared zoospore production over time in two samples of a single Bd isolate having different passage histories: one maintained in artificial media for more than six years (JEL427-P39), and one recently thawed from cryopreserved stock (JEL427-P9). In a common garden experiment, we then exposed two different amphibian species, *Eleutherodactylus coqui* and *Atelopus zeteki*, to both cultures to test whether Bd attenuates in pathogenicity with *in vitro* passages. The culture with the shorter passage history, JEL427-P9, had significantly greater zoospore densities over time compared to JEL427-P39. This difference in zoospore production was associated with a difference in pathogenicity for a susceptible amphibian species, indicating that fecundity may be an important virulence factor for Bd. In the 130-day experiment, *Atelopus zeteki* frogs exposed to the JEL427-P9 culture experienced higher average infection intensity and 100% mortality, compared with 60% mortality for frogs exposed to JEL427-P39. This effect was not observed with *Eleutherodactylus coqui*, which was able to clear infection. We hypothesize that the differences in phenotypic performance observed with *Atelopus zeteki* are rooted in changes of the Bd genome. Future investigations enabled by this study will focus on the underlying mechanisms of Bd pathogenicity.

## Introduction

The fungal pathogen *Batrachochytrium dendrobatidis* (Bd) causes the skin disease chytridiomycosis in susceptible amphibians [1] and has contributed to the decline and extinction of amphibian species worldwide [2]. As a result, studies on this pathogen and many host species have increased greatly since Bd was described in 1998 [3]. Laboratory experiments in particular have provided insights into the differential susceptibility of host species to chytridiomycosis [4,5], the role of environmental factors in disease dynamics [6,7], methods of disease transmission [8,9], pathogenicity of different Bd strains [10-12], and the mechanism by which Bd causes amphibian mortality [13]. 

As with other pathogens, conducting laboratory experiments with Bd requires maintaining the pathogen in artificial media, because isolating a new strain for each experiment is often not feasible or desirable. Studies in which host amphibians are intentionally infected with Bd often assume that *in vitro* cultures retain the properties of wild isolates, particularly the ability to infect hosts and cause disease [14]. Several studies have shown that Bd isolates differ in pathogenicity for amphibian hosts in controlled laboratory exposure experiments [10-12,15], and others have demonstrated that Bd isolates differ in phenotypic characters possibly linked to virulence [15,16]. However, it is not clear whether these differences are a result of variable environmental conditions faced by the isolates in nature or of different *in vitro* management and time since isolation [10]. Recent work has shown that the *in vitro* passage history of Bd strains can affect phenotypic traits potentially linked to pathogenicity [17].

Attenuated pathogenicity is common in artificially maintained cultures of fungi pathogenic to insects, plants, and humans [18,19], yet some fungal species remain pathogenic even after dozens of passages [18]. The question of whether Bd attenuates in culture has received little attention until recently, and it has been unclear whether, and to what degree, Bd attenuates in culture [20]. Brem et al. [14] showed that an artificially maintained Bd isolate became more pathogenic in subsequent exposures after it was passed through an amphibian host, suggesting that Bd virulence attenuates in culture. However, these authors used a Bd strain originating from *Lithobates pipiens* to infect and then re-isolate Bd from *Scaphiopus holbrooki*, introducing a variable (i.e., the *Scaphiopus* epidermis) unrelated to *in vitro* versus *in vivo* maintenance that could have accounted for greater pathogenicity in *S. holbrooki* in subsequent exposures. The conservative interpretation is that Brem et al. demonstrate phenotypic plasticity in Bd performance, which is a notable result. 

 We became concerned about attenuation of Bd following experiments yielding unexpected results. *Eleutherodactylus coqui* experimental frogs expected to be susceptible to infection following high doses of Bd either did not become infected or cleared their infections rapidly. We hypothesized that the Bd isolate used had attenuated in pathogenicity during 6 years of *in vitro* maintenance. Virulence is an emergent property of host, pathogen, and environment [21], and our study design was constructed to tease apart this integrated triangle of cause and effect. To that end, our approach differs from Brem et al. [14] in several important ways. First, we used two samples of the same Bd strain (JEL427), one that was cryopreserved upon isolation and one maintained as active culture since 2005. Next we examined variation in a phenotypic trait, zoospore production, between these two cultures of the same Bd isolate with different passage histories [16]. Zoospore production has been a focus of other studies comparing Bd isolates [15,16,22] and the effects of passage history on a single isolate [17]. This trait is also important because there is an intuitive, mechanistic link with pathogenicity: more zoospores should increase opportunities for transmission between hosts and re-infection of an individual host [23]. 

Finally, we exposed these two cultures to the amphibian species from which JEL427 was isolated, *Eleutherodactylus coqui*, and a second species, *Atelopus zeteki*, under identical environmental conditions. *E. coqui*, a terrestrial direct-developing frog native to Puerto Rico, typically carries low to moderate Bd-infections where this strain is enzootic [24], succumbs to chytridiomycosis in the laboratory [25], and appears to be experiencing disease-related declines at mid-elevations in eastern Puerto Rico [26]. *Atelopus zeteki* is a stream-associated frog with larval development endemic to Panama. Bd-related declines in the wild are common in this species [27,28] and it is also highly susceptible to chytridiomycosis in the laboratory [6,29]. Including *A. zeteki* in the study design yields a common garden experiment, a framework for testing the performance of two cultures of the same Bd isolate with different evolutionary histories under conditions in which the treatment environment and host were held constant (a “common garden”). This control over two elements of the virulence triangle allowed us to be more confident that differences in Bd performance resulted from variation in the third element of the triangle—the pathogen—and explore the possibility that variation in Bd’s performance between two cultures of the same strain with different histories reflected genetic changes in Bd rather than phenotypic plasticity. 

## Methods

### Ethics statement

Our research strictly followed the guidelines of and was approved by the Arizona State University Institutional Animal Care and Use Committee, the University of Maryland Institutional Animal Care and Use Committee, and the Maryland Zoo in Baltimore Institutional Animal Care and Use Committee. We obtained permission to collect and export *E. coqui* frogs from the Departamento de Recursos Naturales y Ambientales in Puerto Rico (permits 2009-IC-015, 2009-IC-014) and the Department of Land and Natural Resources in Hawaii (permit EX10-08). 

### Strain phenotype experiment

Bd produces motile zoospores that infect amphibian skin. Zoospores encyst on the skin surface and develop into thalli within the skin. The mature zoosporangia then cleave into new zoospores after 3-5 days [30]. To test if passage history yielded measurable phenotypic differences in Bd isolate JEL427, we first compared zoospore production over time in two different samples of this single isolate [17]. JEL427 was isolated in 2005 from an infected *E. coqui* frog collected in the El Yunque forest of eastern Puerto Rico. Some of this original culture was cryopreserved [31], and some of the culture was transferred into new solutions of 1% tryptone broth every 3-4 months and maintained at 4°C at the University of Maine. In 2010, we received some of the JEL427 maintained as active culture since 2005, because the cryopreserved sample was initially unavailable. We subsequently maintained it at 18°C for more than a year, passing the culture into 1% tryptone broth monthly. This culture, which experienced a total of at least 39 passages between the two laboratories, will be hereafter referred to as “JEL427-P39”. 

In 2011, a sample of the cryopreserved JEL427 held at the University of Puerto Rico was revived by PAB for this experiment and transferred into 1% tryptone broth. We then passed it into new 1% tryptone broth monthly and maintained it at 18°C. This culture, which experienced roughly 9 total passes, will be hereafter referred to as “JEL427-P9”. Thus, these two cultures are the same age, originating in 2005 from the same source isolate, but have different passage histories. The selection pressure on the JEL427-P9 culture was effectively halted during cryopreservation.

Although the temperature at which Bd isolates are maintained in artificial media can influence zoospore production [20,22], we do not consider the prior maintenance of JEL427-P39 at a colder temperature to be a concern for our study, because we maintained the culture at 18°C for at least 15 months before using it in the strain-phenotype and frog-exposure experiments. This procedure should have provided enough time for the JEL427-P39 culture to acclimate to the warmer temperature.

For the strain-phenotype experiment, we grew the JEL427-P39 and JEL427-P9 cultures on 1% tryptone agar plates using week-old broth culture. We sealed plates and incubated them at 18°C for 5-6 days, and on inoculation day, we flooded plates with 2 ml of sterile 1% tryptone broth and allowed them to sit for 30 minutes while the zoosporangia released zoospores into solution. We then collected the solution from each plate using a sterile pipette and allowed it to drain through a sterile #4 cone-style coffee filter lining a glass funnel, which allows zoospores to pass through but not zoosporangia [32]. Using separate filters and funnels for each culture to prevent cross-contamination, we collected the zoospore filtrate for each culture into separate 50 ml sterile tubes.

We quantified the zoospore concentration of each filtrate using a hemacytometer (Hausser Scientific ® Bright-Line), making two concentration counts of total zoospores for each culture and averaging them. We then diluted the stock solutions to an identical starting concentration of 10^5^ zoospores/ml and transferred 1 ml of stock solution for each culture into 10 ml of 1% tryptone broth in sterile BD Falcon™ 25 cm^2^ tissue culture flasks. This procedure yielded 5 replicate flasks of the JEL427-P39 culture and 5 replicate flasks of the JEL427-P9 culture, which were maintained at 18°C.

We quantified zoospore density for 12 consecutive days for each replicate flask, making two concentration counts for each replicate per day using both chambers of the hemacytometer and averaging them. The same researcher (PFL) made concentration counts each day to be consistent, but we randomized the order of flasks to be counted and kept them unknown to the counter to avoid bias. We replicated the experiment a month later to ensure repeatability of results and this time counted both motile and total zoospores for each replicate daily. We compared zoospore density over time using repeated-measures ANOVA and conducted analyses using JMP^®^ 10 software.

### *Eleutherodactylus coqui* exposure experiment

Although the passage history of Bd strains is thought to cause measurable phenotypic changes in culture [17,22], it is unknown if these changes correspond to a difference in pathogenicity for an amphibian host. To answer this question, we collected 36 adult common *coqui* frogs (*Eleutherodactylus coqui*, mean snout-vent length [SVL]=41.2 mm) from El Yunque, Puerto Rico and near Hilo, Hawaii to use in an exposure experiment (18 frogs per site). We captured frogs at night using plastic bags worn as gloves to prevent cross-contamination with Bd zoospores. In the laboratory we swabbed frogs using sterile fine-tipped cotton swabs (Medical Wire & Equipment 113) to determine Bd infection status [33] and weighed, measured, and sexed each animal. 

The frogs in this study served as uninfected controls in a previous experiment by PFL, so all animals had been in captivity for approximately one year at the beginning of the experiment. We maintained the laboratory at 21°C on a 12-hour day-night cycle and housed frogs individually in 5.7 liter Sterlite plastic storage boxes lined with damp sphagnum moss. Each container had a water dish to maintain humidity and *Cecropia* leaves for refugia. We transferred frogs to new terraria with clean sphagnum moss bi-weekly. Terraria were sterilized with a bleach solution and rinsed three times before re-use. Moss was autoclaved and rinsed thoroughly for re-use a maximum of three times, at which point it was discarded and new moss was used. We fed frogs vitamin-dusted crickets *ad libitum* twice a week and misted terraria daily. 

All frogs were confirmed to be Bd-negative at the beginning of the study. We extracted DNA from skin swabs using PrepMan Ultra and analyzed samples using the standard real-time quantitative polymerase chain reaction (qPCR) assay [34] modified by Hyman and Collins [35]. We gave frogs unique identification numbers and randomly assigned them to three treatment groups: exposure to JEL427-P39 (n=12), exposure to JEL427-P9 (n=12), and exposure to a sham solution (control, n=12).

For the Bd exposure, we grew both the JEL427-P9 and JEL427-P39 cultures on 1% tryptone agar plates for 5-7 days, flooded them with 1% tryptone broth, and filtered the resulting liquid to obtain a pure zoospore stock solution, as described above. We determined zoospore concentration by counting motile zoospores in both chambers of the hemacytometer and averaging the counts. We diluted the stock solutions with purified water to obtain a concentration of 1x10^5^ zoospores/ml for both cultures. The sham solution for the control group consisted of an equivalent amount of 1% tryptone broth (without zoospores) diluted with purified water. During inoculation, we placed frogs in 236 ml plastic cups with lids containing 10 ml of the JEL427-P9 culture, the JEL427-P39 culture, or the sham solution for 10 hours on two consecutive days, using a new pair of nitrile gloves whenever a different frog was handled to prevent cross-contamination.

We swabbed and weighed frogs every ~15 days and monitored morbidity and mortality daily for 80 days. To minimize suffering of moribund frogs, we euthanized them by immersion in 300 mg/l tricaine mesylate (MS-222) when their righting reflex was lost. With both *E. coqui* and *A. zeteki*, we found that loss of righting reflex was the only reliable indicator of imminent death. The most important dependent variable in our study was survival time, and we may have biased our experimental results if we had euthanized frogs sooner as they began exhibiting milder clinical signs of chytridiomycosis (e.g., darkening pigmentation). At the end of the experiment, we euthanized surviving frogs with MS-222 and donated sham-infected frogs to the National Aquarium in Baltimore. 

To compare the average infection intensity over time between frogs exposed to JEL427-P9 and JEL427-P39, we designed a mixed effects model with first order autocorrelation using the package ‘nlme’ in R [36,37]. Infection intensity data were log-transformed prior to analysis. We treated time and passage history as fixed effects and individual frog ID as a random effect. We analyzed a second model that included an interaction between time and treatment in the model. 

### *Atelopus zeteki* exposure experiment

We conducted a second exposure experiment using Panamanian golden frogs (*Atelopus zeteki*, mean SVL=46.5 mm), a species known to be highly susceptible to chytridiomycosis in the lab [6,29] and the wild [28]. We obtained 70 captive-bred *Atelopus zeteki* frogs, 15 months post-metamorphosis, from the Maryland Zoo in Baltimore with support of Project Golden Frog. Although this species is Critically Endangered [38], captive-bred surplus animals were approved for scientific research. 

We allowed frogs to acclimate to laboratory conditions for one month. Animal husbandry was identical to that described for *Eleutherodactylus coqui*, with the following exceptions: we fed frogs fruit flies or small crickets every other day, placed frogs into new clean terraria with sterilized moss weekly, and used plastic refuges instead of leaves. 

We randomly assigned frogs to three treatment groups as in the previous experiment: exposure to JEL427-P39 (n=30), exposure to JEL427-P9 (n=30), and exposure to a sham solution (control, n=10). We undertook this experiment in two phases, 5 weeks apart, with the treatment groups subdivided evenly, e.g. JEL427-P39 (phase 1: n=15, phase 2: n =15). We conducted the experiment twice to ensure repeatability of results. Since *Atelopus zeteki* is highly susceptible to chytridiomycosis, we gave frogs in the exposure groups a lower dose of Bd than in the *E. coqui* experiment (10^2^ zoospores/ml in both phases) [29]. As in the *E. coqui* experiment, we exposed frogs to Bd zoospores in 236 ml plastic cups for 10 hours on two consecutive days. We swabbed and weighed frogs every ~15 days and monitored morbidity and mortality daily for 130 days. At the end of the experiment, we reserved surviving frogs for 30 days to use in a subsequent experiment by KRL. 

## Results

### Strain phenotype experiment

**Figure 1:**
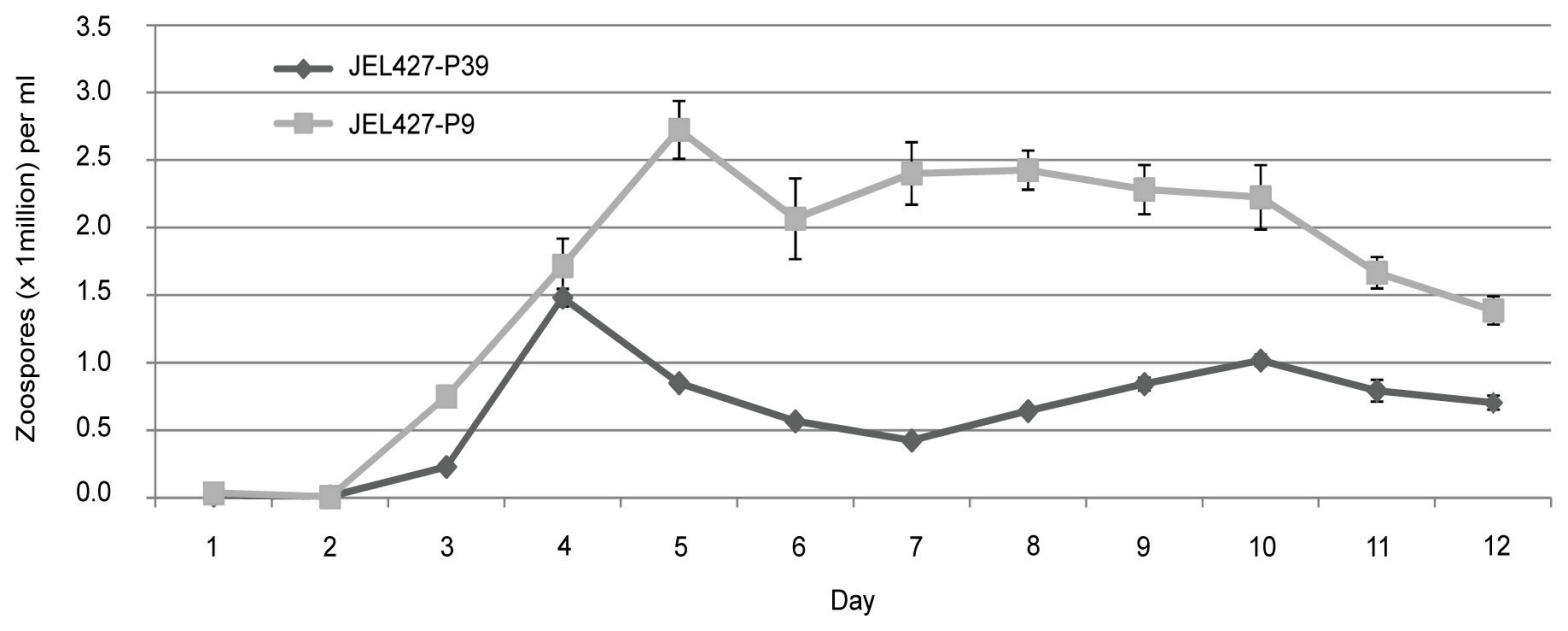
Mean zoospore concentration over 12 days for JEL427-P9 and JEL427-P39. These two cultures of the same Bd strain have different passage histories: JEL427-P9 was cryopreserved upon isolation and passed ~9 times, while JEL427-P39 was maintained in vitro for 6 years and passed ~39 passes. Error bars represent standard error.

We combined data for both strain phenotype experiments, since the only difference between them was a six-week difference in start date. The JEL427-P9 culture showed significantly greater zoospore density (zoospores/ml) over time (repeated measures ANOVA, p < 0.0001; Figure 1) compared to the JEL427-P39 culture. The trends were nearly identical when counts were made using motile zoospores instead of total zoospores (data not shown).

### *Eleutherodactylus coqui* exposure experiment

Given the results of the strain phenotype experiment, we hypothesized that the higher zoospore output in the younger culture would yield greater pathogenicity in susceptible amphibians. Specifically, we predicted that JEL427-P9 would lead to higher Bd infection prevalence, infection intensity, and frog mortality than JEL427-P39.

**Figure 2:**
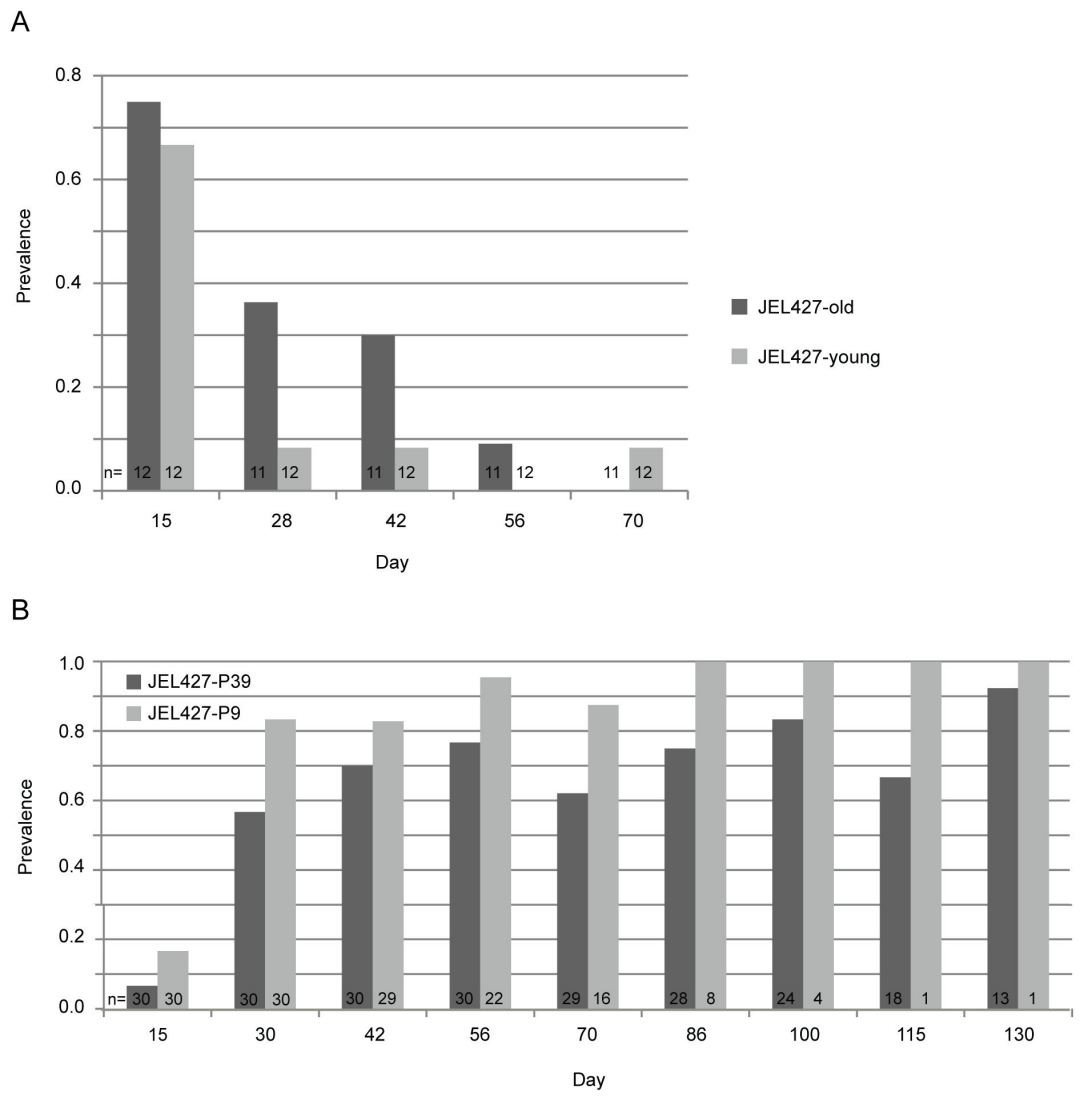
Prevalence of Bd infection in (a) *Eleutherodactylus coqui* and (b) *Atelopus zeteki* exposed to JEL427-P9 or JEL427-P39.

**Figure 3:**
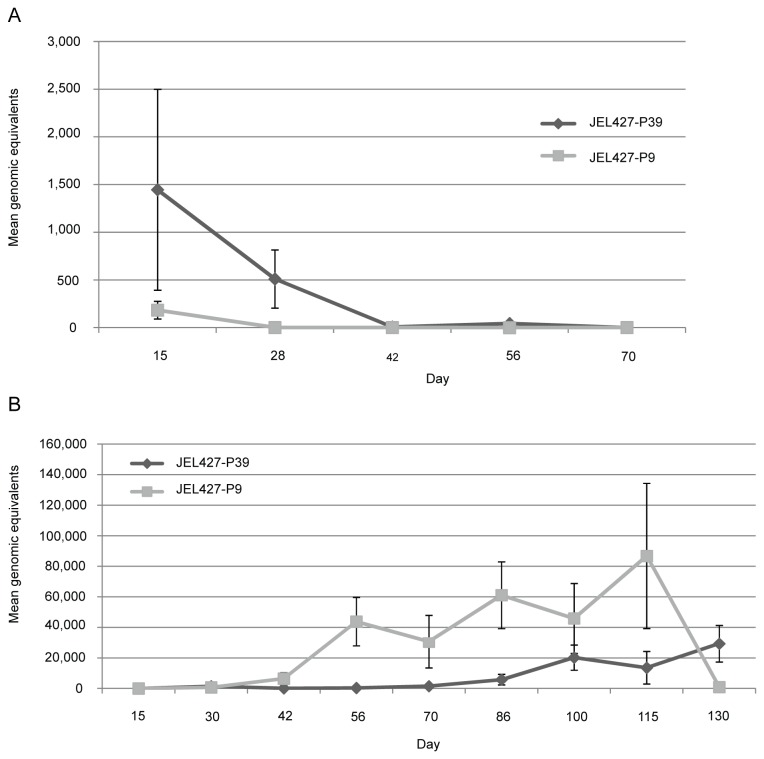
Mean infection intensity for (a) *Eleutherodactylus coqui* and (b) *Atelopus zeteki* frogs exposed to JEL427-P9 or JEL427-P39.

On day 15 post-exposure, infection prevalence among *E. coqui* frogs was 67% in the JEL427-P9 group and 75% in the JEL427-P39 group, indicating that not all frogs became infected during exposure (Figure 2a). Prevalence after day 15 dropped sharply, as frogs started clearing infection despite the large exposure dose. The results of the mixed effects model indicate no significant difference in mean infection intensity (Figure 3a) over time between the two groups (-0.937 log genomic equivalents, p=0.15). Finally, there was no significant difference in mortality between the three treatment groups, including the controls (Log-rank test, p=0.37). Only 1 frog died during the experiment, from the JEL427-P39 group. The frog may have succumbed to chytridiomycosis given its relatively high pathogen load (12,816 zoospore genomic equivalents), but all other Bd-exposed frogs cleared infection within 80 days. 

### *Atelopus zeteki* exposure experiment

Our results with *E. coqui* led us to repeat the experiment with a more susceptible species, *Atelopus zeteki*, which we predicted would more readily become infected upon initial exposure to Bd. Given the much lower Bd dose used in the *A. zeteki* experiment, it took longer for infections to build to detectable levels. Most *A. zeteki* frogs yielded Bd-negative swabs at day 15 but were positive on day 30, indicating a large proportion of false negatives up through day 15 (Figure 2b). This suggests that researchers may be significantly under-sampling early or low-level Bd infections in the lab and field. Eventually, all but one Bd-exposed *Atelopus*, in the JEL427-P39 group, tested positive during the course of the experiment, while none of the control frogs became infected. By day 86, Bd prevalence among surviving frogs in the JEL427-P9 group reached 100%.

The mixed effects model in which time and passage history were treated as fixed effects revealed that *A. zeteki* frogs exposed to JEL427-P9 experienced higher average infection intensity over time compared to the JEL427-P39 group (3.22 log genomic equivalents, p<0.01; Figure 3b). Including the treatment*time interaction decreased the model’s AIC by 1, indicating only slight improvement. 

**Figure 4:**
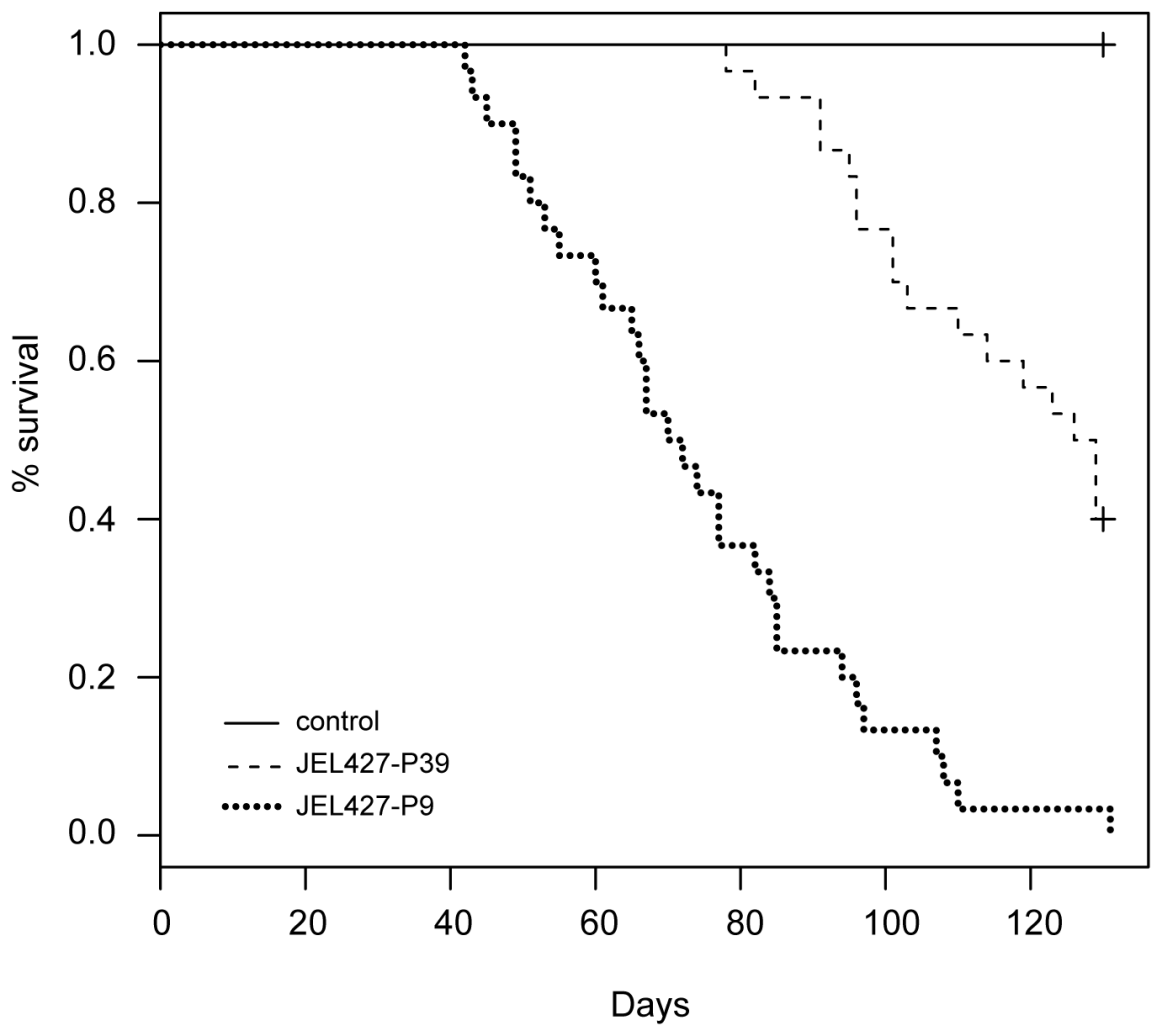
Survival pattern for *Atelopus zeteki* frogs exposed to JEL427-P9 (n=30), JEL427-P39 (n=30), or a sham solution (n=10, control).

Survival rate differed markedly between JEL427-P9 and JEL427-P39 groups. Most Bd-exposed *A. zeteki* eventually showed clinical signs of chytridiomycosis including skin sloughing, darkening pigmentation, weight loss, abnormal posture, lethargy, and ultimately loss of righting reflex, at which point frogs were euthanized. None of the *A. zeteki* frogs exposed to the JEL427-P9 culture survived (0/30), while 40% of the frogs exposed to the JEL427-P39 culture survived (12/30) the 130 day experiment (Figure 4). However, all but one of the frogs exposed to JEL427-P39 were Bd-positive at the end of the experiment and eventually died while waiting to be used in a subsequent experiment. The JEL427-P9 frogs experienced shorter survival times following inoculation than JEL427-P39 frogs (Log-rank test, p < 0.0001), and all control frogs (10/10) survived the full 130 days.

## Discussion

Concern about the possible degeneration of artificially-maintained Bd isolates has surfaced relatively recently [14,15,17], as researchers, including ourselves, noticed that some Bd cultures no longer infected or caused disease in amphibian subjects [14]. Attenuation is well known in fungi pathogenic to insects, plants, and humans, but other species of fungi show no loss of pathogenicity after dozens of passages [18]. We combined strain phenotype and amphibian exposure experiments using a single Bd isolate to demonstrate that our experimental Bd isolate attenuated in pathogenicity when maintained in artificial media. *A. zeteki* frogs exposed to a sample of JEL427 cryopreserved upon isolation experienced greater infection intensity over time, died sooner, and had 100% mortality in the 130 day experiment, compared with frogs exposed to a sample of the same isolate with a longer *in vitro* passage history. This effect was not apparent in *E. coqui*, a species that is currently persisting with low to moderate Bd-infections where this strain is endemic [24], as all but one frog survived and cleared infection across both treatments. 

This difference in Bd pathogenicity for *A. zeteki* was associated with phenotypic differences between the two cultures, specifically zoospore production over time. Voyles [17] demonstrated that passage history can affect the zoospore production of a single Bd isolate and hypothesized that this might translate into variation in pathogenicity based on an isolate’s history. Other fungal pathogens exhibit reduced sporulation when maintained in artificial media [18,19,39]. We predicted that the JEL427 culture with the shorter passage history would have higher fecundity for two reasons. First, evolution of JEL427-P9 was effectively halted during cryopreservation, making it more likely that this culture retained the infective and pathogenic capabilities of wild type Bd. Second, we transferred our cultures into new media monthly, when zoospore output was low due to diminishing food supply. In contrast, Voyles [17] transferred cultures into new media weekly when zoospore density was high, likely selecting for greater zoospore output over time. Our *in vitro* strain phenotype experiments showed that the JEL427-P9 culture indeed produced significantly more zoospores/ml over time than the JEL427-P39 culture. 

Our data showing that average infection intensity in *A. zeteki* frogs exposed to JEL427-P9 was significantly greater for the JEL427-P39 group further suggests that zoospore production is an important component of Bd pathogenicity. In studies with other susceptible species, individual amphibians with the greatest pathogen loads are more likely to succumb to chytridiomycosis [13,40]. However, factors that co-vary with increased zoospore production may have led to the greater pathogenicity of the JEL427-P9 culture. Sporangium size, rather than zoospore production, was associated with pathogenicity of Bd isolates in a previous study [15]; we did not quantify sporangium size. A recent study of the entomopathogenic fungus *Beauveria bassiana* showed that attenuation in artificial media was characterized by both reduced sporulation and a decline in the activity of a spore-bound alkaline serine protease, which is known to be important in penetration of insects and subsequent pathogenicity [19]. Genomic analyses have uncovered expansions in protease gene families in Bd relative to other fungi, leading researchers to hypothesize that proteases play an important role in Bd pathogenicity [41,42]. More work is needed to resolve the relationship between zoospore production and Bd pathogenicity. 

Because it is not clear how long it takes for Bd to attenuate in culture, or even which strains will or will not attenuate, freshly isolated Bd or cryopreserved stock should be used in amphibian exposure experiments [14], especially those designed to test for susceptibility to Bd. Our experiments with *A. zeteki* and *E. coqui* showed that artificially maintained isolates may still kill frogs, but only if the species is highly susceptible.

Our work further highlights the importance of choosing an appropriate amphibian host species in Bd challenge experiments. Our first frog exposure experiment, with *E. coqui*, shed no light on the effect of passage history on Bd pathogenicity. All but one *E. coqui* frog cleared infection within 60 days after exposure to a high dose of Bd, despite being the original source species for JEL427. This finding suggests that species surviving an epidemic can develop resistance or tolerance to the original source of an infection, but may still be vulnerable if a new strain is introduced. Conversely, in the exposure experiment with *A. zeteki*, a difference in pathogenicity between the cultures with different passage histories was evident. This species shows high susceptibility to Panamanian Bd strains in the field [28] and lab [29], and it proved highly susceptible to JEL427 from Puerto Rico. 

Most importantly, our research lays the groundwork for future investigations into the mechanisms of Bd virulence. We hypothesize that the differences in phenotypic performance observed in this study are rooted in changes of the genome. Maintenance in artificial media may impose intense selection pressure on genes regulating production of zoospores or spore-bound proteases. It has recently been shown that the genome of Bd changes very rapidly during passage [43]. Whole genome sequencing will enable comparisons between a pathogenic and an attenuated version of our isolates [41,42], potentially providing insights into the genetic determinants of pathogenicity and help explain the genetic variation and performance among Bd lineages [12,44]. Better understanding of the mechanisms of Bd pathogenicity will strengthen our ability to manage a disease having widespread and often devastating impacts on amphibian species [40,45] and communities [46]. 
